# Results of spinal accessory nerve to suprascapular nerve transfers in children with brachial plexus birth injury

**DOI:** 10.1177/17531934241276372

**Published:** 2024-09-14

**Authors:** Maria Hyttinen, Henrikki Rönkkö, Pasi Paavilainen, Mika Helminen, Jarkko Jokihaara

**Affiliations:** 1 Faculty of Medicine and Health Technology, Tampere University, Tampere, Finland; 2 Division of Musculoskeletal Diseases, Tampere University Hospital, Tampere, Finland; 3 Pihlajalinna Hospital, Tampere, Finland; 4 Tays Research Services, Tampere University Hospital, Wellbeing Services County of Pirkanmaa, Tampere, Finland and Faculty of Social Sciences, Health Sciences, Tampere University, Tampere, Finland

**Keywords:** Brachial plexus, birth injury, shoulder joint, external rotation, nerve transfer

## Abstract

Shoulder external rotation after brachial plexus birth injury can be restored by transfer of the spinal accessory nerve to the suprascapular nerve, or more distally to its infraspinatus branch. We studied the outcome of these nerve transfers in 52 patients with a minimum postoperative follow-up of 12 months (mean 7.3 years). The median postoperative improvement in shoulder external rotation was 120° (interquartile range [IQR] 45–135) after anterior and 110° (IQR 83–120) after dorsal spinal accessory nerve transfer to the suprascapular nerve main trunk, and 110° (IQR 80–125) after transfer to the infraspinatus branch. Patients operated after 20 months obtained external rotation ≥90° less frequently. The results of this study suggest that a decision about distal nerve transfer for shoulder external rotation is recommended at 1.5 years of age.

**Level of evidence:** III

## Introduction

Brachial plexus birth injuries (BPBI) occur as a result of a difficult delivery in 0.9–5.1 per 1000 births (DeFrancesco et al., 2019; Hoeksma et al., 2004; Mollberg et al., 2023). The majority of injuries affect the upper trunk (roots C5 and C6), which results in poor or absent shoulder and elbow movements (Boome and Kaye, 1988). Suprascapular nerve (SSN) branches from the upper trunk and the route of SSN through the suprascapular notch and its relationship to bony structures makes the nerve vulnerable to traction injuries (Shin and Spinner, 2005). The infraspinatus muscle is innervated by the SSN and is the most important external rotator of the shoulder (Hughes and An, 1996). Active external rotation (ER) is usually reduced (Pondaag et al., 2005) and is one of the last movements to recover in patients with BPBI (Hoeksma et al., 2004).

The standard primary operative treatment is neuroma resection and reconstruction with sural nerve grafts. If that is not indicated, or in patients with an upper root avulsion injury, peripheral nerve transfers have increasingly been used. Sometimes nerve reconstruction is carried out along with a peripheral nerve transfer (Wells et al., 2022). Peripheral nerve transfers have several advantages over proximal reconstruction with grafts; for example, a shorter distance for the regenerating nerve fibres to reach the target muscle (Addas and Midha, 2009; Fox and Mackinnon, 2011) and a more distal dissection outside the area of the initial injury with less risk of losing other functions (Fox and Mackinnon, 2011). Shoulder ER can be restored by transfer of the spinal accessory nerve (SAN) to the SSN (SAN–SSN) (Pondaag et al., 2005; Seruya et al., 2015) or more distally to the infraspinatus branch of the SSN (SAN–SSN-IB) leaving the nerve branch to supraspinatus muscle intact (Kaiser et al., 2021; Sommarhem et al., 2015).

In this study, we have analysed the results of SAN–SSN and SAN–SSN-IB. The aims of the present study were to assess the improvement in ER, whether one of the techniques results in better improvement and how age at surgery is associated with the outcome.

## Methods

**Figure 1.**
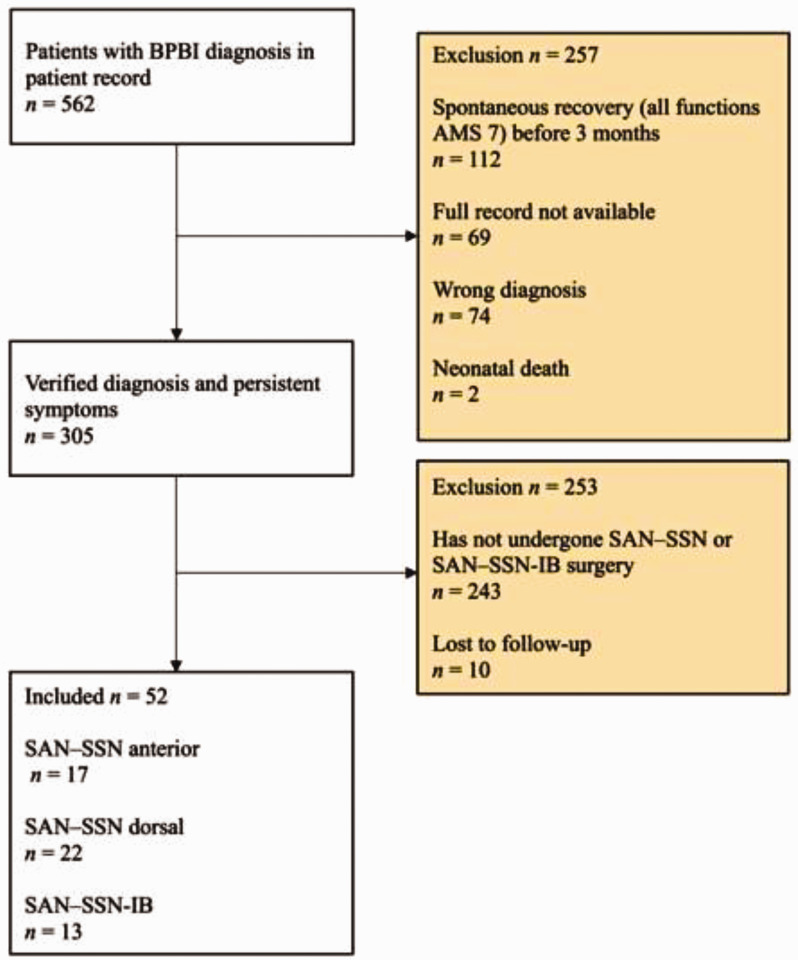
Inclusion and exclusion of patients.

We screened all patients with BPBI born between 2002 and 2020 and treated at Tampere University Hospital, Finland, which serves as a tertiary referral centre for a population of 2 million. Patients were screened from electronic records using the diagnosis codes for birth injuries to the peripheral nervous system (ICD-10: P14.0, P14.1, P14.2, P14.3, P14.8, P14.9). We excluded patients with a wrong diagnosis, incomplete patient data and those who had recovered normal upper extremity function (all movements: Active Movement Scale [AMS] 7 (Curtis et al., 2002)) within the first 3 months of life. Our final study cohort (*n* = 52) included all the patients with BPBI who had not recovered ER and who had undergone SAN–SSN or SAN–SSN-IB with a minimum postoperative follow-up of 12 months. A total of 10 patients were lost to follow-up (Figure 1).

In our clinical practice during the study period, most patients with BPBI who underwent nerve transfer surgery were referred to the BPBI clinic before the age of 3 months. Our treatment protocol included repeated systematic clinical assessment and ultrasound examination of the shoulder. If we observed restricted passive ER or signs of posterior subluxation of glenohumeral joint in the ultrasound before the age of 1.5 years, we used botulinum toxin-A (BTX) injections to the subscapularis, pectoralis major and teres major/latissimus dorsi muscles, and a shoulder spica in ER for 4–6 weeks. Lack of active ER at 1.5 years was considered to be an indication for SAN–SSN. If passive ER was restricted after the age of 1.5 years, we carried out subscapular tendon lengthening with resection of the coracoid process and anterior capsule release if needed. Some patients with insufficient improvement in ER later underwent teres major to infraspinatus tendon transfer surgery.

We collected information about symptoms and clinical findings at different time points, Narakas grade at 1 month of age (Al-Qattan, 2003), details about nerve transfer surgery, including age at nerve transfer surgery, type of nerve transfer (SAN–SSN or SAN–SSN-IB), preoperative and postoperative range of motion in ER and details about any other operations. The time of the last follow-up for clinical findings was the last clinical assessment in the patient record or, if the patient had undergone a secondary tendon transfer operation for ER, the last examination before the operation.

The range of active and passive movement in shoulder ER was assessed independently by a surgeon (PP, HR or JJ) and by a paediatric physiotherapist. ER was determined as the angle between the sagittal plane and the long axis of the forearm with the arm in full adduction and the elbow in 90° of flexion, within a range of −90° to 90°. Postoperative improvement in active shoulder ER was calculated by comparing the preoperative range of active movement and active movement at the final examination. Most of the patients (*n* = 48/52) had no active ER preoperatively (forearm in −90° internal rotation). We defined clinically meaningful improvement of the active range of motion as 90°, in which the forearm can reach the sagittal plane when the arm is in adduction and the elbow is in flexion, making it possible to reach the mouth without a trumpet sign (shoulder abduction compensating for insufficient ER when bringing the hand to the mouth). This is equal to AMS 6, i.e. at least half of the range of movement (Curtis et al., 2002). Because data regarding improvement in the active range of ER had a bimodal distribution (modes 0° and 110°), we categorized the improvement in ER into two classes to carry out the analysis: 90° improvement in ER resulted in active movement to the neutral position (AMS 6) and was regarded as the limit between the two groups (improvements of <90° and ≥90°).

SAN–SSN operations were done through a dorsal incision along the spine of the scapula (Bahm et al., 2005) or through an anterior supraclavicular incision, if done at the same time as brachial plexus exploration or reconstruction. In SAN–SSN, the SAN is transferred end-to-end to the SSN and to the SAN near the suprascapular notch of scapula, and in SAN–SSN-IB the SAN is transferred to the SSN-IB near the spinoglenoid notch (Sommarhem et al., 2015). In our treatment protocol, SAN–SSN-IB is used if the patient is able to abduct the arm above 90° (horizontal level). We determined the reinnervation and recovery of the affected muscles by measuring active ER.

### Statistical analysis

Improvement in ER, defined as the difference between the preoperative and postoperative ranges of motion, was tested separately for both study groups (SAN–SSN and SAN–SSN-IB) using the Wilcoxon signed-rank test. Binary logistic regression was used to determine how the type of surgery (anterior or dorsal SAN–SSN or SAN–SSN-IB) influences improvement in active ER. We first created separate crude models with the type of surgery and age at surgery as predictors for improvement of ER ≥90°. Because all the SAN–SSN-IB operations were carried out on patients aged over 17 months and all anterior SAN–SSN operations before 12 months, and the age at nerve transfer operation may have an overall impact on muscle recovery via the duration of muscle denervation, we created an adjusted model with surgery type and age at surgery. Contribution of the variables to the model was assessed using ANOVA.

We used three age groups based on clinical treatment practice: early operations before 12 months of age; operations at 12–20 months of age; and late-term operations after 20 months of age. Patients operated on before 12 months had undergone brachial plexus reconstruction and simultaneous SAN–SSN or isolated SAN–SSN only if the finding in brachial plexus exploration was root avulsions. If early operative treatment was not indicated, the decision regarding SAN–SSN or SAN–SSN-IB was made after a systematic follow-up until 18 months of age. If ER was still absent, the operation took place few weeks after the decision. In the last age group (after 20 months), the patients with BPBI had been referred either for a first clinical assessment later than usual or the primary brachial plexus reconstruction had not led to recovery of ER, and SAN–SSN or SAN–SSN-IB surgery was carried out later.

Data are presented as mean with standard deviation (SD) for normally distributed variables or as median with weighted interquartile range (IQR) for variables with skewed distribution. Statistical analyses were carried out using R version 4.3.2 (R Foundation for Statistical Computing, Vienna, Austria). *p*-values are reported for Wilcoxon signed-rank tests. For logistic regressions, results are presented as crude odds ratios (COR) or adjusted odds ratios (AOR) with 95% confidence intervals (CI) and *p*-values. *p*-values <0.05 were considered statistically significant throughout the study.

## Results

**Figure 2.**
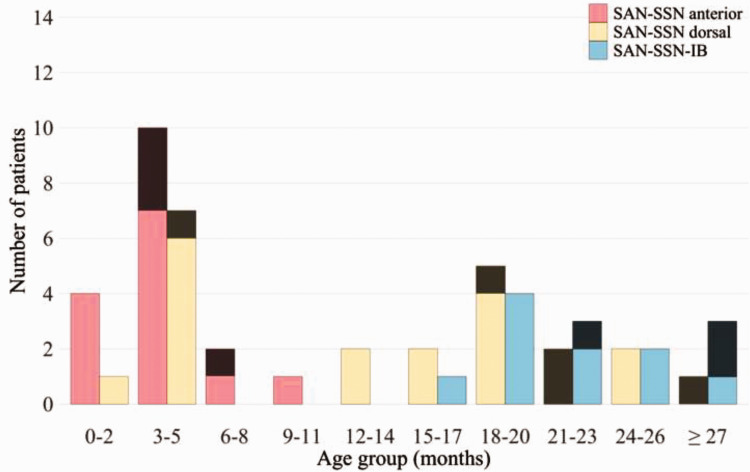
Number of spinal accessory to suprascapular nerve (SAN–SSN) anterior and dorsal approach and spinal accessory to infraspinatus branch of suprascapular nerve (SAN–SSN-IB) transfer surgeries at different age groups clustered by surgery type. Bars are stacked by the obtained improvement in active shoulder external rotation (ER), which is dichotomized into improvement of <90° and ≥90°. Striped sections of the bars indicate patients with improved ER <90°.

Our consecutive study cohort included 52 patients with BPBI operated on between 2002 and 2023. The numbers of patients treated by each procedure and their preoperative Narakas grading are shown in Table 1. Their ages at surgery are shown in Table 2 and Figure 2. The median age at surgery and the median follow-up are shown in Table 3.

**Table 1.** Narakas classification (Al-Qattan, 2003) and affected nerve roots based on clinical findings at 1 month of age.

Narakas classification at 1 month	SAN–SSN anterior (*n* = 17)	SAN–SSN dorsal (*n* = 22)	SAN–SSN-IB (*n* = 13)	All (*n* = 52)
Narakas 1 (C5–C6)	–	3	1	4
Narakas 2 (C5–C6–C7)	8	19	8	35
Narakas 3 (C5–C6–C7–C8–T1)	2	–	3	5
Narakas 4 (C5–C6–C7–C8–T1 + Horner)	6	–	–	6
Not reported	1	–	1	2

SAN–SSN: spinal accessory nerve to suprascapular nerve transfer; SAN–SSN-IB: spinal accessory nerve to infraspinatus branch of suprascapular nerve transfer.

**Table 2.** Binary logistic regressions comparing probability of ER improvement ≥90° between surgeries and age groups.

Category	Patients (*n*)	COR (95% CI)	*p*-value (crude)	AOR (95% CI)	*p*-value (adjusted)
Surgery			0.973		0.998
SAN–SSN anterior	17	1		1	
SAN–SSN dorsal	22	1.0 (0.2 to 4.7)	0.953	2.2 (0.3 to 46.6)	0.527
SAN–SSN-IB	13	1.0 (0.2 to 6.2)	0.977	6.7 (0.3 to 263.2)	0.243
Age at surgery (months)			**0.049**		**0.022**
>20	25	1		1	
12–20	14	11.1 (1.5 to 233.8)	**0.041**	16.7 (1.9 to 416.0)	**0.029**
<12	13	3.4 (0.8 to 15.7)	0.099	12.0 (1.0 to 334.4)	0.073

*p-*values in bold are statistically significant.

AOR: adjusted odds ratio, model including both surgery and age at surgery; CI: confidence interval; COR: crude odds ratio; SAN–SSN: spinal accessory nerve to suprascapular nerve transfer; SAN–SSN-IB: spinal accessory nerve to infraspinatus branch of suprascapular nerve transfer.

**Table 3.** Age at surgery, follow-up, postoperative outcome and secondary surgery.

	SAN–SSN (anterior) (*n* = 17)	SAN–SSN (dorsal) (*n* = 22)	SAN–SSN-IB (*n* = 13)	All (*n* = 52)
Age at surgery (months)	4 (3–5)	17 (5–20)	22 (20–27)	14 (5–21)
Postoperative follow-up (years)	7.1 (3.4)	10.0 (4.2)	3.5 (2.0)	7.3 (4.3)
Improvement in ER (°)	120 (45–135)	110 (83–120)	110 (80–125)	110 (90–128)
Other (secondary) surgery, by age (months)				
Anterior release or subscapular tendon lengthening				
<12	5	–	–	5
12–20	–	1	–	1
>20	–	2	1	3
Tendon transfer surgery for ER				
<12	3	1	–	4
12–20	1	–	–	1
>20	–	2	1	3
Humeral rotational osteotomy				
<12	1	–	–	1
12–20	–	–	–	–
>20	–	–	–	–

Data are presented as mean (SD) or median (IQR).

ER: active shoulder external rotation in adduction; IQR: interquartile range; SAN–SSN: spinal accessory nerve to suprascapular nerve transfer; SAN–SSN-IB: spinal accessory nerve to infraspinatus branch of suprascapular nerve transfer; SD: standard deviation.

The median postoperative improvement after each procedure is shown in Table 3. Statistically significant improvements in ER occurred after anterior SAN–SSN (*p* < 0.001), after dorsal SAN–SSN (*p* < 0.001) and after SAN–SSN-IB (*p* = 0.001). The median active range of ER at the final assessment was 110° (from −90° to 20°) after both dorsal SAN–SSN and SAN–SSN-IB operations and 120° (from −90° to 30°) after anterior SAN–SSN. Improvement in active ER ≥90° was observed in 13/17 patients who underwent anterior SAN–SSN, in 17/22 patients who underwent dorsal SAN–SSN and in 10/13 patients who underwent SAN–SSN-IB (Figure 2). The effect of surgery type on outcome was non-significant in the crude model, as well as in the model adjusted for age at surgery (Table 2).

Of the patients operated before 12 months, at 12–20 months and after 20 months, 20/25, 13/14 and 7/13 patients showed an improvement of ≥90° in active ER, respectively (Figure 2). Age was found to be a significant predictor for ER improvement in the crude model and in the adjusted model (Table 2). Patients operated after 20 months were used as a reference group in the subgroup analyses and patients operated earlier were more likely to improve ER ≥90° (Table 2).

The patients had a passive range of ER from −90° to ≥45° at the time of the surgery, except for five patients who had restriction of passive ER (mean −90° to 14°; SD 12), who underwent a subscapular tendon lengthening or anterior release of the glenohumeral joint capsule at the same procedure. Four patients underwent a release after SAN–SSN, with passive ER range from −90° to 0°, and three of them recovered active ER after the release. Eight patients underwent tendon transfer surgery at a mean age of 54 months (SD 25) because active ER after nerve transfer was still insufficient (Table 3). All these patients had no active ER before the tendon transfer; five of them underwent lengthening of the subscapular tendon or a release in the same procedure, owing to restricted passive ER (mean −90° to 18°; SD 10).

## Discussion

We measured an improvement in shoulder ER after peripheral nerve transfer surgery in 52 patients with BPBI. SAN–SSN via anterior and dorsal approaches and SAN–SSN-IB resulted in good and similar improvement in ER. Patients operated on earlier gained ≥90° postoperative improvement in ER more frequently than those operated on after 20 months. Our findings support the practice of systematic early assessment of shoulder function in patients with BPBI and, in the absence of ER, decision making about nerve transfer surgery by the age of 1.5 years.

The use of peripheral nerve transfers is a gaining popularity (O’Grady et al., 2017; Wells et al., 2022). To our knowledge, there have been no previous studies comparing the outcomes of SAN–SSN and SAN–SSN-IB. The strength of this study is that it was an unselected consecutive cohort of patients with an established treatment protocol and comprehensive systematic assessments, and a long-term follow-up. Clinical assessments were carried out independently by a physician and a paediatric physiotherapist, which increases the reliability. Nevertheless, the difficulty of grading joint movements in infants and small children may cause inaccuracy in the results. Owing to the relatively low incidence of BPBI, the sample sizes in some of the analyses were small, which reduces the statistical power.

When the outcomes of the anterior and dorsal approaches for SAN–SSN have been compared in adult patients, similar or better improvement in ER has been reported using the dorsal approach compared to the more traditional anterior approach (Maurya et al., 2021; Souza et al., 2014). In addition, exploring the SAN through an anterior approach may lead to partial denervation of the upper trapezius muscle (Bhandari and Deb, 2011). We used an anterior approach in patients who had a simultaneous brachial plexus exploration or reconstruction, mainly at the beginning of the study period before a dorsal approach became the standard practice.

Overall, the improvement of ER after SAN–SSN and SAN–SSN-IB was good. This is in agreement with previous studies on SAN–SSN (Abdelmaksoud et al., 2020; Gmeiner et al., 2015; Ruchelsman et al., 2010; Seruya et al., 2015; van Ouwerkerk et al., 2006) and SAN–SSN-IB (Grahn et al., 2020; Sommarhem et al., 2015). Some studies suggest SAN–SSN results in better ER than brachial plexus reconstruction with nerve grafts (Manske et al., 2020; Seruya et al., 2015; Smith et al., 2020). In contrast, Pondaag et al. (2005) reported that only 10 of 21 patients who underwent SAN–SSN achieved active true glenohumeral ER movement from −90° to 0°, and there was no difference in improvement in ER between SAN–SSN and grafting from C5 to SSN. A limitation in our analysis was that the youngest patient who underwent SAN–SSN-IB was operated on at the age of 17 months, so differences between SAN–SSN-IB and SAN–SSN operations in young children cannot be determined based on this study. In previous studies, the youngest patients have been aged 1.5 years at SAN–SSN-IB surgery (Grahn et al., 2020; Sommarhem et al., 2015) and there are no data available on outcomes of early SAN–SSN-IB operations.

There has been a wide variety of age groups in previous studies of the outcomes of SAN–SSN operations in paediatric patients. In the study by Ruchelsman et al. (2010), patients were divided into two groups by age at surgery ≤9 months and >9 months; with this classification, the age at surgery did not influence the outcome. Van Ouwerkerk et al. (2006) found no difference in the improvement of ER in operated children who were younger or older than 12 months or younger or older than 18 months. In the study by Gmeiner et al. (2015), the mean age at SAN–SSN surgery was 3.7 years, which is later than usual in the care of children with BPBI; however, the improvement of active ER after surgery was good. In contrast, Abdelmaksoud et al. (2020) reported that results were worse if the patient was operated on after the age of 16 months. In our study, the majority of patients who were operated on before the age of 20 months, according to our treatment protocol, showed an improvement in ER of ≥90° but the proportion was smaller after the age of 20 months. It is important to acknowledge the limitations of the small number of patients in each group, which increases the uncertainty of our results.

The optimal age for making the decision about SAN–SSN or SAN–SSN-IB, should be considered against the expected probability of spontaneous recovery, which decreases over time. Our results support the view that follow-up for spontaneous recovery of ER can be continued until the age of 1.5 years without compromising the outcome of peripheral nerve transfer surgery to obtain shoulder ER. Prolonged denervation of the shoulder muscles may lead to muscle imbalance, an internal rotation contracture and glenohumeral dysplasia (Grahn et al., 2019; Mascarenhas et al., 2014; Pöyhiä et al., 2010); therefore, the follow-up protocol must include daily passive ROM exercises, ultrasound screening for glenohumeral subluxation, and BTX injections and splinting in ER, if needed (Grahn et al., 2022). However, if the patient is first examined at a later age, some improvement in ER may still be achieved with nerve transfer surgery.
